# A protocol for a randomised active-controlled trial to evaluate the effects of an online mindfulness intervention on executive control, critical thinking and key thinking dispositions in a university student sample

**DOI:** 10.1186/s40359-016-0122-7

**Published:** 2016-04-12

**Authors:** Chris Noone, Michael J. Hogan

**Affiliations:** School of Psychology, NUI Galway, University Road, Galway, Ireland

**Keywords:** Mindfulness, Critical thinking, Thinking dispositions, Executive functioning, Executive function, Dual processes

## Abstract

**Background:**

While most modern research focuses on the clinical benefits of mindfulness, an emerging body of work suggests that mindfulness can facilitate self-regulation of everyday thinking in typically developing individuals. This behaviour is best captured using critical thinking assessments. The aim of this paper is to describe a rigorous, pre-registered study which will investigate the effect of an online mindfulness intervention on Executive Functioning, critical thinking skills and associated thinking dispositions.

**Method:**

The design employed is a randomised-controlled 2 (condition) X 2 (time) parallel-group design which is explanatory in nature. A sample of at least 60 participants will be recruited from the pool of students at NUI Galway, with those between the ages of 18 and 65 with an adequate level of English included. Participants will be randomly assigned following screening, using block randomisation with a fixed block of 6 and a 1:1 ratio, to either the mindfulness meditation group or a sham meditation group. Both groups will be given access to the Headspace app. This is an app which provides guided meditations to users. Participants in each group will receive unique codes granting access to either the experimental or active-control intervention materials. Group allocation will be double-blinded. The primary outcome measures will assess mindfulness, executive functioning, critical thinking, actively open-minded thinking and need for cognition. Secondary outcome measures will assess eudaimonic and hedonic wellbeing, positive and negative affect, and real-world outcomes. These will be measured at baseline and at the end of the intervention. Manipulation checks will assess adherence to the intervention, meditation quality and task difficulty and enjoyment.

**Discussion:**

If this intervention proves effective, it will show the potential of mindfulness practice to facilitate everyday critical thinking and should stimulate more interest in this line of research. If ineffective, claims regarding mindfulness and thinking skills should be tempered. This research was funded by a Galway Doctoral Research Scholarship awarded to the first author and was facilitated by Headspace Inc. who provided the intervention materials. The trial is registered in the ISRCTN registry and any protocol amendments will be recorded there (RCT ID: ISRCTN16588423. Registered 7th January 2016).

## Background

In a world where we have more information than ever before available to us, it is vital to be able to analyse this information, evaluate its quality, relevance, credibility, and logical soundness and apply it in appropriate circumstances [1]. This ability is often described as critical thinking [2]. In psychological literature, critical thinking is considered a metacognitive process involving skills such as analysis, evaluation and inference that, when used appropriately, increase the chances of producing a logical conclusion to an argument or solution to a problem [3]. Developing these thinking skills is important in order to make the most of the information available to us rather than just passively assimilating it [4]. Critical thinking is recognised as an important higher-order cognitive process which requires a non-automatic response to a problem situation in order to avoid the inappropriate application of heuristics and biases [5]. The demand for skill in critical thinking has made the question of what determines effective critical thinking an essential one to investigate. Research has focused on types of instruction such as critical thinking infusion and immersion [6], dispositional factors such as cognitive effort [7], open-mindedness [8] and truth-seeking [2], and cognitive ability [5]. Investigations into the importance of thinking dispositions and cognitive ability converge in research on the self-regulation of higher-order cognition [9]. Recently, mindfulness, a state of attention involving non-reactive awareness of present moment internal and external stimuli, has also been a target of research and has been suggested as beneficial for critical thinking [10, 11]. Previous intervention studies have shown improved performance in aspects of critical thinking following mindfulness training but have not examined the underlying mechanisms (e.g. [12–16]). A recent cross-sectional study found evidence for inhibition mediating a positive relationship between mindfulness and critical thinking [17].

In considering the self-regulation of critical thinking, mindfulness is a functionally relevant construct. Though conceptualisations of mindfulness vary, all highlight the role of mindfulness in enhanced self-regulation of thought and emotion and all contrast mindful information processing with automatic, habitual or heuristic information processing, often referred to as mindlessness [18]. The link between mindfulness and self-regulatory processes can best be explored by considering the currently most cited operational definition of mindfulness in light of cognitive models of self-regulation [19]. Notably, in cognitive models of self-regulation, the mobilisation of self-regulatory resources is characterised by the effective operation of the executive functions (EFs) that support and govern working memory [20]. These processes also regulate attention and are integral to the process of of mindfulness which consists of two components: present-moment attentional focus coupled with non-reactive monitoring of one’s ongoing experience [21]. Practitioners of mindfulness meditation cultivate a state of non-reactive present-moment attention by focusing their attention on the present-moment, usually using an anchor such as their breath, and paying full attention to any internal or external stimuli that arise while supressing the elaboration of affective cues triggered by these stimuli [22]. From time to time, the attention of the practitioner will wander, at which point they should notice this and bring their attention back to the present moment [23].

There is an emerging consensus that EF involves three basic processes: updating, inhibition and shifting [24]. These processes are engaged during the practice of mindfulness and empirical studies have shown that regular mindfulness practice can enhance these processes [10, 23, 25, 26]. Updating refers to the active revision and monitoring of thinking [27]. During a mindful state, the act of focusing in the present moment requires constant updating of working memory as internal and external stimuli change [21, 28]. The updating and maintenance of working memory is a vital self-regulatory process as it facilitates the accurate active representation of goals and goal-related information [20]. Inhibition refers to the active, deliberate suppression of thoughts or responses and the maintenance of attention on goal-relevant information [24, 27]. Inhibition is involved in keeping attention focused on the present moment by inhibiting elaboration of and reactivity to affective cues. This allows for the early engagement of emotion regulation before intense emotional reactivity to the attended thoughts, feelings and sensations can occur [22]. Shifting can refer to flexibility in use of different strategies to achieve a goal or flexibility in switching between multiple goals [20]. This is engaged during a mindful state when the mind wanders and attention must be directed back to the present-moment. Furthermore, cognitive flexibility has been shown to increase as a result of mindfulness practice using tasks such as the Stroop [19, 28, 29] and the Hayling task [30].

Each of these EF processes of updating, inhibition and shifting support higher-order cognitive processes involved in problem-solving [31], metacognition [32] and decision-making [33]. In fact, it has been suggested that the operation of working memory by EFs is the key mechanism through which higher-order cognition is engaged. The default interventionist dual-process theory of higher-order cognition posits two distinct types of cognitive processing. Type-1 processing generates intuitive, automatic responses by default. Type-2 processing allows further reflective processing but requires the engagement of EF which may or may not intervene depending on the individual and context [34]. Therefore, critical thinking depends on Type-2 cognitive processes which depend on EF. An emerging body of theoretical and empirical work has linked mindfulness with enhanced executive functioning and certain types of higher-order cognition related to critical thinking, including insight problem-solving [12, 13], moral reasoning and ethical decision-making [14–16].

Importantly, each of these studies suggested (but did not examine) that mindfulness facilitated the interruption of automatic responses and allowed more reflective processing, consistent with default interventionist theory.

It has been claimed that mindfulness should facilitate critical thinking in higher-education, based on early Buddhist conceptualisations of mindfulness as clarity of thought [11]. There is clearly theoretical support and some empirical evidence for this claim but it is important to test the veracity of this claim in the most rigorous way available. The most rigorous way to test the effects of mindfulness is using randomised-controlled intervention studies which compare mindfulness training to a control condition. This design has been used to test the effects of Mindfulness-based Stress reduction, Mindfulness-based Cognitive Therapy and focused mindfulness meditation training programmes on health, wellbeing and cognitive outcomes (see reviews – [10, 35–37]). However, for the most part, the control groups employed have involved waitlist controls rather than an active-control condition [38]. Still, this is an improvement from initial studies which were often non-randomised or lacked any control groups or even manipulation checks [10]. An active-control group is desirable in order to rule out the potential effects of relaxation, received attention and demand characteristics. Strategies for designing an active-control condition have included using audiobooks [39], using progressive muscle relaxation training [40] and using sham meditation training. Sham meditations are the most sophisticated approach but also the least used due to their relatively recent development. They involve breathing exercises which are introduced to participants under the label of mindfulness practice. It is important that these exercises are guided by the same facilitator and for the same amount of time as the guided mindfulness practice which the experimental group engage in. Therefore, the one key difference between the two groups is the nature of the instructions given. Where the active-control group are instructed to “continue breathing as we sit in meditation” every few minutes, the experimental group are given clear instructions on how to pay attention to their breathing in order to cultivate a mindful state [39].

Advances in technology are allowing the design of mindfulness interventions with more experimental control than previously possible [41]. The development of smartphone and web applications focused on the delivery of guided meditations in particular has made it easier to include active control conditions, objectively measure time spent meditating and reduce the resources needed for running an intervention as well as the demands placed on the participants. Previous studies involving smartphone delivery of mindfulness interventions focused on workplace stress [42], wellbeing [41], depression [43] and compassion [44]. Each of these studies showed comparable results to previous traditional interventions focused on the same outcome variables and can be considered more rigorous due to the standardisation of instruction across participants in the experimental group, the inclusion of active-control materials which participants expected to benefit from in the same way as those in the experimental condition, and objective measures of adherence to the intervention (provided through the app) rather than self-report.

### The current study

This protocol paper describes the development of an intervention which makes use of the Headspace mindfulness meditation app for smartphones, tablets and web browsers to evaluate its effects on executive control, critical thinking and key thinking dispositions as well as the real-world outcomes of critical thinking. This intervention was developed in order to test, in the most rigorous way available, hypotheses developed as a result of careful review of the literature on the effects of mindfulness on self-regulation and cognitive abilities and studies carried out previously by the authors suggesting that mindfulness may facilitate more effective critical thinking. The rationale for this intervention relies heavily on a specific type of dual-process theory known as the default-interventionist theory of higher-order cognition [34], which can be used as a framework to integrate research on the effects of mindfulness on attention, executive function and self-regulation of behaviour in general and research on the self-regulation of higher-order cognition, such as critical thinking [17].

In summary, the central research question here is: does regular mindfulness meditation practice facilitate critical thinking through the enhancement of executive function? To answer this, the proposed study aims to ascertain whether a 6-week online mindfulness meditation intervention increases trait mindfulness, executive function, critical thinking performance and endorsement of key critical thinking dispositions to a greater extent than an active-control sham meditation condition. The hypotheses to be tested can be seen in Table 1. This aim will be achieved by testing these hypotheses using the measures and analyses described in the next section. It is also intended to investigate the role of executive function in mediating the predicted positive relationship between mindfulness and critical thinking performance. Finally, the study aims to explore the participants’ experiences of taking part in an online mindfulness meditation intervention and the real-world outcomes they perceived.

**Table 1:** Study hypotheses

Outcomes	Variable	Measure		Hypothesis	Analysis
Primary	Mindfulness	Five Facet Mindfulness Questionnaire	1	Mindfulness will increase more for the mindfulness meditation (MM) group than for the sham meditation (SM) group from t_1_ to t_4_	Mixed ANOVA
	Critical Thinking	Halpern Critical Thinking Assessment^1^, Heuristic and Biases items^2^	2	Critical thinking will increase more for the MM group than for the SM group from t_1_ to t_4_ *(a* ^*1,2*^ *)* and this effect will be moderated by baseline endorsement of thinking dispositions *(b* ^*1,2*^ *)*	Mixed ANOVA, ANCOVA
	Thinking Dispositions	Actively Open-minded Thinking^1^, Need for Cognition^2^	3	Endorsement of critical thinking dispositions will increase more for the MM group than for the SM group from t_1_ to t_4_ *(a* ^*1,2*^ *)*	Mixed ANOVA
	Executive Control	Sternberg Working Memory Task	4	Executive control will increase more for the MM group than for the SM group from t_1_ to t_4_ *(a)* and this increase will mediate the relationship between levels of mindfulness and critical thinking performance following the intervention *(b)*	Mixed ANOVA, SEM
Secondary	Wellbeing	Warwick-Edinburgh Mental Wellbeing Scale	5	Wellbeing will increase and negative affect will decrease more for the MM group than for the SM group from t_1_ to t_4_	Mixed ANOVA
	Positive Affect and Negative Affect	Positive Affect and Negative Affect Schedule subscale	6	Positive affect will increase more for the MM group than for the SM group from t_1_ to t_4_ *(a)*	Mixed ANOVA
	Real-world Outcomes	Real-world Outcomes Inventory	7	Negative real-world outcomes will decrease more for the MM group than for SM group from t_1_ to t_4_	Mixed ANOVA
Manipulation Checks	Meditation Quality	Practice Quality-Meditation	8	Meditation quality will be positively associated with increases in mindfulness *(a)*, executive control *(b)* and critical thinking *(c* ^*1,2*^ *)* and meditation quantity *(d)*, task enjoyment *(e)* and task difficulty *(f)* and it will be higher in the MM group and across time.	Correlation, Mixed ANOVA
	Meditation Quantity	Total Minutes Spent Meditating	9	Meditation quantity will be positively associated with increases in mindfulness *(a)*, executive control *(b)* and critical thinking *(c* ^*1,2*^ *)* and meditation quality *(d)*, task enjoyment *(e)* and task difficulty *(f)* and will not differ across time or groups.	Correlation, Mixed ANOVA
	Task Enjoyment	Technology Acceptance Model Questionnaire subscale	10	Task enjoyment will be positively associated with increases in mindfulness *(a)*, executive control *(b)* and critical thinking *(c 1,2*) and meditation quality *(d)*, meditation quantity *(e)* and task difficulty *(f)* and will not differ across time or groups.	Correlation, Mixed ANOVA
	Task Difficulty	Technology Acceptance Model Questionnaire subscale	11	Task difficulty will be positively associated with increases in mindfulness *(a)*, executive control *(b)* and critical thinking *(c 1,2*) and meditation quality *(d)*, meditation quantity *(e)* and task difficulty *(f)* and will not differ across time or groups.	Correlation, Mixed ANOVA
	Intervention Acceptability	Items from Kirkpatrick et al. [60]	12	Intervention acceptability will be positively associated with increases in mindfulness *(a)*, executive control *(b)* and critical thinking *(c* ^*1,2*^ *)* and meditation quantity *(d)*, task enjoyment *(e)* and task difficulty *(f)* and it will be higher in the MM group but will not differ across time.	Correlation, Mixed ANOVA
	Attrition	No. of participants lost from baseline to t_4_	13	Attrition will be negatively associated with meditation quality *(a)*, meditation quantity *(b),* task enjoyment *(c)* and task difficulty *(d)* and will not differ across time or groups.	Correlation, Mixed ANOVA

## Methods/design

### Design

The CONSORT guidelines for evaluation of randomised controlled trials [45], the CONSORT extension for non-pharmacological treatment interventions [46], the SPIRIT checklist of protocol items and the TIDIER checklist for intervention description and replication [47] were adhered to in the design of this study and this protocol paper.

This study involves a two-arm randomised-controlled superiority trial with one intervention condition, guided mindfulness meditation, and one active-control condition, sham meditation. The design employed is a 2 (condition) X 2 (time) parallel-group design which is explanatory in nature. Measurement will take place at baseline (i.e. before randomisation, T1) and 6 weeks after baseline (T4). The delivery of the content of both the intervention condition and the active-control condition will take place between T1 and T4. Manipulation checks will be carried out to assess intervention acceptability, technology acceptance and meditation quality 2 weeks after baseline (T2) and 4 weeks after baseline (T3). See Table 2 for a description of the procedure.

**Table 2:**
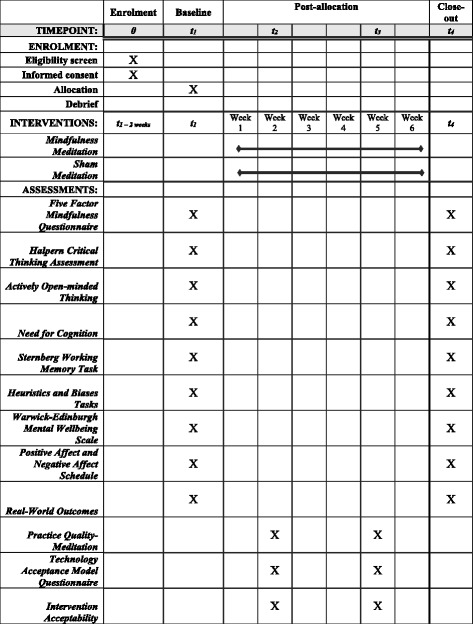
Timeline of Intervention

### Sample size (Incl. flow chart)

The statistical program G*Power was used to conduct power analysis in order to determine the appropriate sample size. Adhering to Cohen’s [48] guidelines for small (*r* = 0.1), medium (*r* = 0.3), and large (*r* = 0.5) effects, two-tailed alpha of .05 was assumed for all tests. With 2 groups, 6 measurements, an assumed correlation among repeated measures of 0.3 (typically low in such research; [49]) as well as a medium effect size (again typical in research on the cognitive effects of mindfulness; [10]) and a power of 0.8, the recommended sample size for mixed (repeated-measures and between factors) ANOVA was 56. As it is recommended that the sample size be a multiple of the number of measurements, a sample of at least 60 will be sought, 30 per group. Because of an anticipated attrition rate of 20 %, 38 participants are required per group i.e. 78 in total. Previous studies using Headspace have reported attrition rates between 20 and 40 % [41, 44]. We expect an attrition rate closer to 20 % because of the incentives in the form of course credit, lunches provided at data collection and free subscription to Headspace for 6 months following the intervention.

### Sample characteristics

Competent university students over the age of 18 will be invited to participate. Though the study will be open to all students and advertised widely, it is anticipated that the majority of participants will be first and second year psychology students. First year and second year psychology students are awarded credit for participating in a variety of undergraduate final year research projects and postgraduate research projects. The sample will comprise University students at NUI Galway, over 18 years of age and below 65 years of age with English as first language or university level English (i.e. equivalent to 80 on TOEFL or 6.5 on IELTS; both standardised and recognised tests of English as a foreign language).

### Eligibility

Our inclusion criteria specify that University students at NUIG who are over 18 years of age, below 65 years of age and have either English as first language or university level English (i.e. equivalent to 80 on TOEFL or 6.5 on IELTS; both standardised and recognised tests of English as a foreign language) will be eligible for this study.

Those who are alcohol or drug dependent; are currently on any form of sedating medication, have suffered from any medical conditions associated with a head injury, spinal injury, epilepsy, or stroke (because these can interfere with cognitive performance) or do not possess normal or corrected-to-normal vision and hearing (required for computerised tasks) will be excluded. Furthermore those exhibiting clinical levels of depression, anxiety or psychotic symptoms (as assessed with the Modified Mini Screen) will be excluded. Debriefing phone calls will be made to those excluded and they will be offered access to the intervention materials. An experienced clinician has agreed to provide advice on the management of any participant in whom a high level of emotional distress is identified. Any such participants will also be provided with a database of relevant professionals and professional organisations.

### Recruitment and randomisation

Students enrolled in psychology will be invited to participate and on acceptance of this invitation will fill out the screening questionnaire online. Those that satisfy the inclusion criteria will be selected to take part in the intervention and will be randomised to either the mindfulness meditation group or the sham meditation group with a 1:1 ratio. Block randomisation will be employed using a fixed block of 6 [50]. Unique Headspace access codes corresponding to the two conditions will be provided to the researcher. These will be labelled Condition A and Condition B and only after analysis will the nature of these conditions be revealed to the researchers by Headspace. Therefore both participants and researchers will be blinded. However, blinding can be readily undone on a participant-by-participant basis if necessary.

Potential participants will be invited to take part by email and through advertisements on social media. Announcements regarding the study will also be made by the researchers in lectures attended by 1st year and 2nd year undergraduate psychology students.

### Intervention

Intervention materials will be delivered via Headspace, a commercially available application which runs on all major smartphones, tablets and web browsers. The Headspace meditation scripts are designed by an individual with Buddhist monastic training who guides users through mindfulness meditations and key concepts related to mindfulness meditation using both audio and visual materials. In order to participate, individuals are required to have access to a smartphone or desktop computer with Internet access. Headspace makes meditating accessible by combining technology and simple techniques that are designed for new meditators. Participants can practice at any time of day wherever they prefer. Headspace offers straightforward, guided, bite-sized mindfulness training that is non-religious. We have signed guarantees with Headspace that participant data collected through Headspace will never be sold, distributed, or publicised (except anonymously in scientific publications with Headspace having no involvement in the conduct, analysis, or reporting of the research in any way).

Participants will be sent an email introducing Headspace and describing the sign-up process. To get started, participants are required to register on headspace.com using their name and email address. Each participant will be given a unique code providing free access to Headspace for the duration of the study. After registering, participants may begin meditating straight away.

The proposed intervention is 6-weeks in length. All participants will be encouraged to practice meditation/sham-meditation daily for the course of the 6-weeks by listening to each of the 30 ten-minute guided sessions which they will access through the Headspace app. The nature of the sessions they can access depends on the group they are assigned to.

### Experimental condition

Participants in the experimental condition will gain access to 30 sessions of guided mindfulness practice. These sessions introduce the concept and practice of mindfulness training and each session gradually builds on the previous one. The sessions are guided by Andy Puddicombe, a trained Buddhist monk who is also a registered meditation consultant with the UK Health Commission. Each session begins with the participant begin instructed to sit, close their eyes and take deep breaths. Following this, participants are guided through mental body scan exercises intended to cultivate a mindful state which involve practicing focusing attention on present-moment sensations in the body without emotionally elaborating on these sensations. Gradually participants learn to re-direct their attention when the mind wanders and to broaden their present-moment awareness to all current internal and external stimuli. Towards the end of the course of sessions, participants are encouraged to apply this type of awareness to everyday activities.

### Active-control condition

Participants in the active-control condition will gain access to 30 sessions of guided sham meditation practice. These sessions discuss meditation and introduce breathing exercises under the guise of mindfulness practice. However, specific instructions for how to pay attention to the breath or other stimuli are not given. Instead, participants are encouraged to sit quietly, with their eyes closed and every few minutes they are reminded to take deep breaths as they sit in meditation. These sessions are also guided by Andy Puddicombe and accessed in exactly the same way as content in the experimental condition. This approach was taken as it controls for both physiological relaxation and expectations regarding meditation. Other approaches used in previous studies have only controlled for one of these [39]. For example, progressive muscle relaxation only controls for physiological relaxation, while mind wandering inductions only control for expectations regarding meditation. These approaches did improve on previous attention-only and audiobook controls and all of these are a significantly better approach than waitlist controls when possible [10].

### Adherence

Objective adherence data will be collected through the Headspace accounts of the participants. These data will include both the number of sessions initiated and the number of minutes spent listening to the guided sessions. One email and one text message per week will be sent to participants to encourage them to adhere to the intervention.

### Data collection

Primary and secondary measures will be taken at baseline and following the end of the intervention (see Table 2). In terms of primary outcome measures, we will assess critical thinking using the Halpern Critical Thinking Assessment [51] and items from the Heuristics and Biases literature [5], mindfulness with the Five Factor Mindfulness Questionnaire (FFMQ;[52]), thinking dispositions with the Need for Cognition scale [53] and the Actively Open Minded thinking scale [8] and executive function with the Sternberg working memory task [54] presented on Inquisit software [55]. Objective measures of meditation adherence (no. of sessions initiated and completed and no. of minutes spent meditating) will be gathered through the Headspace app. Secondary measures will assess effects on wellbeing in order to compare with previous studies employing Headspace. The Positive Affect and Negative Affect Schedule [56] and the Warwick-Edinburgh Mental Wellbeing Scale [57] will be used. All of the measures except the Sternberg Working Memory Task will be presented using SurveyGizmo. Data Collection will take place during the week preceding the start of the intervention in the PC Suites of the School of Psychology at NUI Galway. Three sessions will be scheduled in order to facilitate attendance at different times, each of which will be able to accommodate up to 30 participants comfortably. A break with food and refreshments will be given half way through the procedure. This data collection approach will be repeated during the week following the end of the intervention.

Manipulation checks will be carried out during weeks 2 and 4 of the intervention and will focus on mindfulness meditation quality and task expectations, enjoyment and difficulty. Mindfulness meditation quality will be assessed using the 6 item Practice Quality- Mindfulness questionnaire [58] while task expectations, enjoyment and difficulty will be assessed using the Technology Acceptance Questionnaire [59] and Intervention Acceptability items [60]. These questionnaires will be administered online using SurveyGizmo through a link which will be distributed by email.

### Primary outcome measures

#### Halpern Critical Thinking Assessment (HCTA; [51])

The HCTA involves 25 real-world situations with closed and open questions based on these situations. These situations involve medical research, social policy analysis and other types of problems encountered in everyday life. Five domains of critical thinking are assessed using the HCTA: Verbal reasoning, argument analysis, thinking as hypothesis testing, likelihood and uncertainty, and decision-making and problem-solving. The test includes 5 sets of questions (one open and one closed) for each of these domains. The scoring guide provides answers for forced-choice questions while open-ended questions are graded according to specific grading prompts (for more detail see [61]). Greater scores are awarded to more accurate and comprehensive answers and total scores can range from 0 to 194 [51]. The internal reliability of the HTCA tends to be adequate [51, 61]. The HCTA has been shown to be sensitive to change in previous intervention studies comparing methods of critical thinking instruction [62, 63].

### Heuristics and Biases items [5]

This series of standard items assessing cognitive biases is included as there is evidence that these assess an aspect of critical thinking not captured by traditional measures [5]. These items assess participants’ ability to deal with problems involving causal base rates, noncausal base rates, the law of large numbers, regression to the mean, the gambler’s fallacy, conjunctions, covariation, Bayesian reasoning, framing and probabilistic reasoning (for full details of the items see [5]). Each of these items will be scored as either correct (1) or incorrect (0). Though these are not representative of a unidimensional construct, it has been shown to be useful to aggregate scores on these items [5].

### Five Factor Mindfulness Questionnaire (FFMQ; [52])

This questionnaire assesses levels of dispositional mindfulness. The FFMQ includes 39 items which tap five facts of mindfulness: describing (i.e. labelling experience with words), observing (i.e. paying attention to sensations, thoughts and feelings), non-reactivity (i.e. noticing thoughts without emotionally responding to them), non-judgment (i.e. acceptance of thoughts and feelings) and acting with awareness (i.e. lack of distraction). It employs a 5-point Likert scale (e.g. 1 = never or very rarely true; 5 = very often or always true). This measure has demonstrated adequate internal consistency and construct validity [64].

### Secondary outcome measures

#### Sternberg working memory task [54]

This task is a measure of executive control of working memory. It involves memorising a series of letters and indicating, as quickly and accurately as possible, whether a probe was in this series or not.

### Positive Affect and Negative Affect Schedule (PANAS; [56])

This scale is the most widely-used instrument for assessing inter- and intra-individual differences in experiences of positive and negative emotion. The PANAS-X consists of 60 items. Each item describes a different feeling or emotion and the scale can be used to assess general levels of positive and negative affect by asking participants to indicate to what extent they felt each of these emotions over the past week using a 5-point Likert scale (e.g. 1 = very slightly or not at all; 5 = extremely). Psychometric evaluations tend to find good reliability for the positive and negative subscales [65].

### Warwick-Edinburgh mental wellbeing scale [57]

This is a 14 item scale of mental well-being covering subjective well-being and psychological functioning, in which all items are worded positively and address aspects of positive mental health. The scale is scored by summing responses to each item answered on a 5 point Likert scale. The minimum scale score is 14 and the maximum is 70. A high score reflects a high level of positive mental health and a low score reflects a low level of positive mental health [66].

### Real world outcomes inventory [62]

This is a behavioural checklist focused on life outcomes from many domains ranging in severity from mildly negative (e.g., paying late fees for a movie rental) to severely negative (e.g., spending a night in jail). It was developed based in the Decision Outcomes Inventory [67]. The version employed here is slightly adapted to ensure cultural relevance. Any items which do not fit the Irish context will not be used (e.g. got blisters from sunburn).

### Potential moderators

#### Need for cognition scale [53]

This unidimensional scale measures the extent to which individuals tend to engage in effortful cognitive activity [53]. The scale includes 18 items which are rated on a 5-point Likert scale (e.g. 1 = extremely uncharacteristic of me; 5 = extremely characteristic of me). It has been extensively validated and has been found to have adequate reliability [68].

### Actively open minded thinking scale [8]

This scale assesses the extent to which individuals tend to approach information in an open and flexible manner as opposed to a rigid manner which leads to resistance to belief change. The scale includes 41 items and these are rated on a 6-point Likert scale (e.g. 1 = strongly agree; 6 = strongly disagree).

### Manipulation checks

In week 2 and week 4 participants will complete short questionnaires assessing mindfulness meditation quality and task expectations, enjoyment and difficulty. These will be completed online, allowing participants to complete them at their convenience. Participants will be asked to complete these measures directly following a meditation session.

### Practice quality- mindfulness questionnaire [58]

This 6 item questionnaire assesses perseverance and receptivity during meditation. Perseverance is defined as the ability to continually redirect attention back to the focus of the meditation. Receptivity refers to the willingness to fully experience what is arising during the course of a meditation session. The items are presented alongside a percentage scale and participants are asked to indicate the percentage of time during their meditation session during which their experience reflected each of the item statements. This scale has been shown to fit a 2-factor structure and has demonstrated a predictive relationship between practice quality and improvements in psychological symptoms [58].

### Technology acceptance model questionnaire (TAM; [59])

Items from the TAM assessing expectations, enjoyment and difficulty using the Headspace app will be presented to participants. The TAM is a widely-used measure of user acceptance of technology. The items on this scale are phrased as statements and are measured on a 5-point Likert scale (e.g. 1 = strongly disagree; 5 = strongly agree).

### Intervention acceptability [60]

Four items assessing satisfaction with the intervention were administered. Two questions using a 5 point Likert scale (e.g. 1 = very dissatisfied; 5 = very satisfied) will assess general satisfaction with the programme and satisfaction with the content of the guided sessions in particular. The next two questions require yes/no answers and relate to whether participants would recommend the programme and whether they felt it was worth their time. Questions like these have been used in previous research examining the acceptability of low-intensity online treatments and across a range of different age groups and health conditions [60].

### Statistical analysis

Data will be primarily analysed through a series of 2 × 2 (time – pre, post x group – mindfulness meditation, sham meditation) mixed ANOVAs for each outcome measure using SPSS. The time x group interaction affects will be assessed in order to investigate differences in between the experimental group and the control group in the amount of change on the dependent variables. Correlations between manipulation check measures will also be examined as will their correlations with FFMQ change scores. AMOS will allow simple mediation analyses to be conducted using Structural Equation Modelling (SEM) to test whether executive function, meditation quality and adherence are mediators of any potential relationship between mindfulness and critical thinking. As noted above, these tests will be adequately powered – including SEM analyses (see [69], for evidence of adequate power for simple mediation using SEM in samples as small as *n* = 30). Our analyses will take an intention-to-treat approach and missing data will be treated with a baseline-observation-carried-forward approach. See Table 1 for specific hypotheses.

### Data management and access

This data management plan has been created using the UCD Data Management Checklist [70]. The data will be saved online through Inquisit (the Sternberg Memory Task) and Surveygizmo (all other tasks and questionnaires). This data is only accessible by the first author. When these data are collated, the second author will also have access to the relevant data files. The data will be saved in both .csv and .sav formats. These files will be stored in encrypted Dropbox folders. A detailed logbook will be created to complement these files. We do not currently have ethical approval to share these data. In accordance with the NUI Galway data retention policy, these data will be retained for 5 years at the NUI Galway School of Psychology (as well as being backed up on Dropbox) and anonymised by replacing student ID numbers and names with randomly generated subject ID numbers.

### Ethics, consent and permissions

This project has received full approval from the NUI Galway Research Ethics Committee [Re. (15/ Sept/03)]. Full written informed consent will be sought from all participants for both their participation and the publication of the results of the research. Participants will be reminded that they are free to withdraw at any time and that their data will be stored securely and anonymously. All data will be stored on password protected hard drives and in accordance with the Data Protection Act. Following completion of data collection, all data will be anonymised. There are no reported risks associated with mindfulness training and similar online mindfulness interventions. The questionnaires, information and activities may highlight a small amount of emotional distress for some people. However, previous intervention studies on mindfulness suggest that only a small number of participants drop out for these reasons [71]. It will be clearly communicated that completing the questionnaires and the intervention is voluntary and that if it does bring up difficulties relevant professionals should be contacted. An experienced clinician has agreed to provide advice on the management of any participant in whom a high level of emotional distress is identified. Any such participants will also be provided with a database of relevant professionals and professional organisations. Adverse consequences of using Headspace have not been reported in previous studies and so we do not have objective criteria for discontinuing the intervention for individuals apart from their own decision to withdraw. The trial is registered in the ISRCTN registry and any protocol amendments will be recorded there (RCT ID: ISRCTN16588423. Registered 7th January 2016).

### Research support

This study is supported with funding awarded to the first author by the NUI Galway Doctoral Research Scholarship. Technical support was provided by Headspace Inc. who provided the infrastructure and content needed to deliver the intervention.

### Dissemination

The results of this study will be reported in the form of a journal article which will be submitted to BMC Psychology upon its completion. Blogs and social media will also be employed by the authors to share the results of this study.

## Discussion

This study aims to investigate the claim that mindfulness practice facilitates critical thinking. It will also test whether executive function mediates the relationship between mindfulness and critical thinking in line with default interventionist theory, previous cross-sectional studies which examined this relationship, and previous experimental studies which suggested this relationship [12–17]. It will achieve this aim by randomising participants to either an experimental condition involving the learning of mindfulness meditation or an active-control condition involving guided sham meditations which will both be delivered through the same online application, Headspace. Dispositional mindfulness, executive function and critical thinking will be assessed at baseline and following the end of the intervention along with measures of intervention adherence, wellbeing, thinking dispositions and real-world outcomes of critical thinking. Manipulation checks assessing intervention acceptability and meditation quality will also be administered.

This study has many strengths. It is pre-registered with the ISRCTN registry which is openly accessible. The use of an active-control which is identical in expectations (which will be measured) and presentation (i.e. through the Headspace application and by Andy Puddicombe, a trained Buddhist monk) represents an advance from the usual attention or waitlist controls employed in previous mindfulness meditation interventions [10]. Another advance is the use of objective measures of intervention adherence. Where most previous studies have relied on self-report measures of adherence (when included), the application used to deliver the intervention materials in this study will also track the amount of guided meditations participants engage in and for how long they engage with them. Finally, participants will be allocated to their respective groups without knowing which was the experimental condition and this information will also be kept from the primary researcher until after data analysis is complete in order to ensure a double-blind RCT design.

There are however some weaknesses associated with this study also. While every effort was made to ensure that the only difference between the guided mindfulness meditations and the guided sham meditations would be the provision of specific instructions to do with building specific mindfulness skills in the experimental condition, it could be that this manipulation makes these guided sessions more engaging and enjoyable than the guided sham meditations. This could lead to differences in attrition rates across the two conditions. Attrition rates in general could be larger than expected which may affect the statistical power of the analyses. Finally, participants will be drawn from the student population only and so any conclusions made based on this study may not be generalizable to the wider population. However, critical thinking is a skill which is of particular importance as an outcome of university education and therefore this study may have practical benefits.

Many companies, universities and other institutions are introducing mindfulness programmes with the promise of improving thinking skills [72]. While there are theoretical and historical reasons supporting this view, it has not been adequately investigated. The significance of this study lies in its rigorous approach to investigating this claim for the first time in the context of an RCT.

### Ethics

This project has received full approval from the NUI Galway Research Ethics Committee [Re. (15/ Sept/03)]. Full written informed consent will be sought from all participants for their participation and the consent form can viewed at this study’s entry in the ISRCTN registry.

### Consent to publish

Though our published results will only feature aggregated group-level information (i.e. no information about specific individuals), full written informed consent will be sought from all participants for the publication of the results of the research.

### Availability of data and materials

We do not currently have ethical approval to share these data. We are happy to provide openly licensed materials and provide information for how to obtain non-openly licensed materials on request.

## Abbreviations

AEA: American economics association
ANOVA: analysis of variance
EF: executive function
FFMQ: five factor mindfulness questionnaire
HCTA: Halpern critical thinking assessment
IELTS: international English language testing system
PANAS: positive affect negative affect schedule
RCT: randomised controlled trial
TOEFL: test of English as a foreign language
